# Supplementary material for: Similar Characteristics of siRNAs of Plant Viruses Which Replicate in Plant and Fungal Hosts
Source: Biology (Basel). 2022 Nov 17;11(11):1672. doi: 10.3390/biology11111672 (PMC9687825; doi:10.3390/biology11111672)
Supplement: Supplementary file 1 [file biology-11-01672-s001.zip › Supplementary Figure S1.pdf]

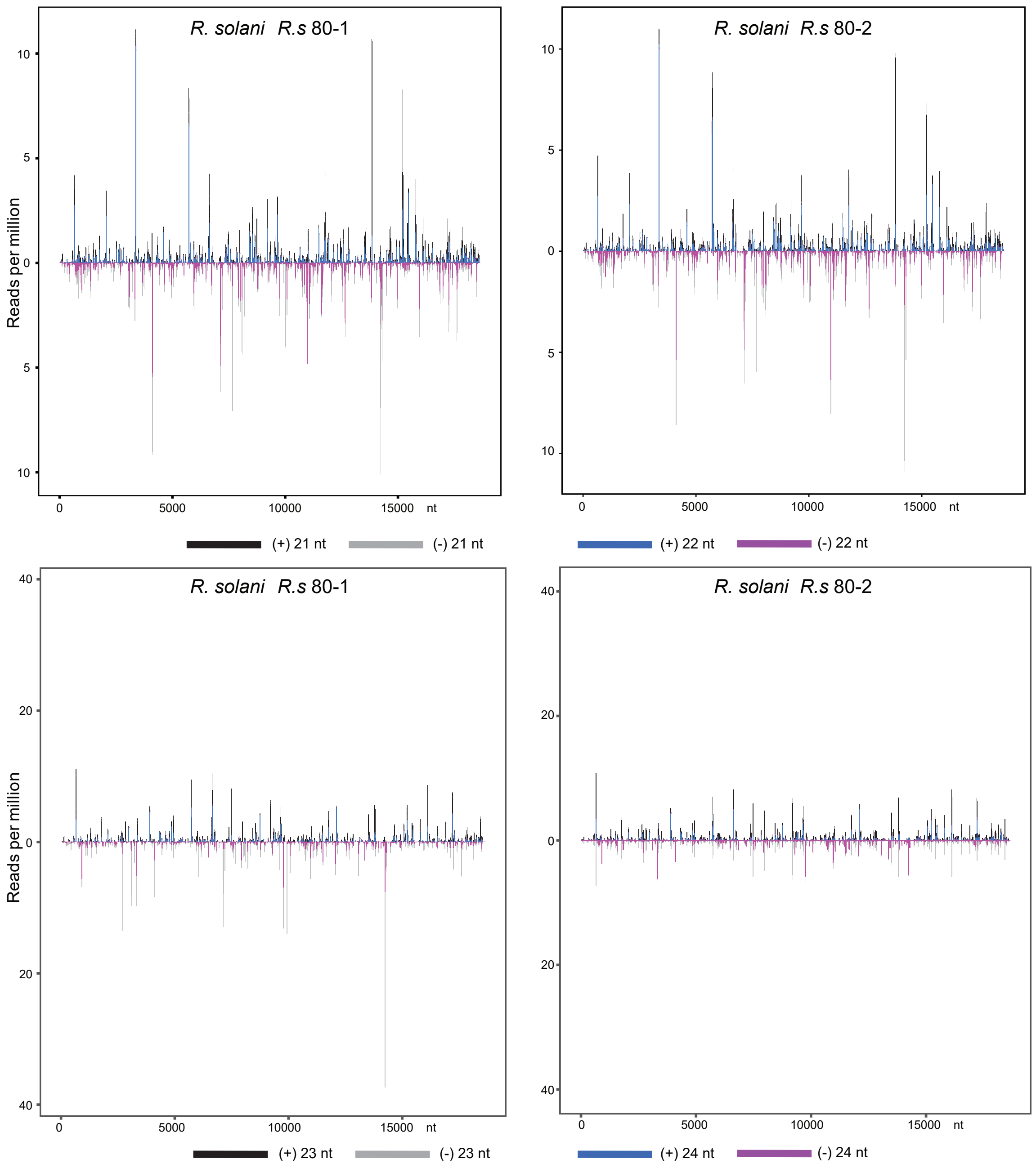

Supplementary Figure S1. Distribution of RsEnLV1 siRNAs (21, 22, 23 and 24 nt) along the viral genome in *R. solani* and *N. benthamiana* libraries. "(−)" and "(+)" indicate siRNAs derived respectively from the complementary (negative) or positive viral genomic strands.
